# Temporal and Spatial Coexistence of Archaeal and Bacterial *amoA* Genes and Gene Transcripts in Lake Lucerne

**DOI:** 10.1155/2013/289478

**Published:** 2013-03-05

**Authors:** Elisabeth W. Vissers, Flavio S. Anselmetti, Paul L. E. Bodelier, Gerard Muyzer, Christa Schleper, Maria Tourna, Hendrikus J. Laanbroek

**Affiliations:** ^1^Department of Microbial Ecology, Netherlands Institute of Ecology (NIOO-KNAW), Droevendaalsesteeg 10, 6708 PB Wageningen, The Netherlands; ^2^Swiss Federal Institute of Aquatic Science and Technology (Eawag), Überlandstrasse 133, 8600 Dübendorf, Switzerland; ^3^Institute of Geological Sciences and Oeschger Centre for Climate Change Research, University of Bern, Zähringerstrasse 25, 3012 Bern, Switzerland; ^4^Department of Aquatic Microbiology, University of Amsterdam, Science Park 904, 1098 XH Amsterdam, The Netherlands; ^5^Department of Genetics in Ecology, University of Vienna, Althanstrasse 14, 1090 Vienna, Austria; ^6^AgResearch Ltd., Ruakura Centre, East Street Private Bag 3115, Hamilton 3240, New Zealand; ^7^Institute of Environmental Biology, Utrecht University, Padualaan 8, 3584 CH Utrecht, The Netherlands

## Abstract

Despite their crucial role in the nitrogen cycle, freshwater ecosystems are relatively rarely studied for active ammonia oxidizers (AO). This study of Lake Lucerne determined the abundance of both *amoA *genes and gene transcripts of ammonia-oxidizing archaea (AOA) and bacteria (AOB) over a period of 16 months, shedding more light on the role of both AO in a deep, alpine lake environment. At the surface, at 42 m water depth, and in the water layer immediately above the sediment, AOA generally outnumbered AOB. However, in the surface water during summer stratification, when both AO were low in abundance, AOB were more numerous than AOA. Temporal distribution patterns of AOA and AOB were comparable. Higher abundances of *amoA *gene transcripts were observed at the onset and end of summer stratification. In summer, archaeal *amoA *genes and transcripts correlated negatively with temperature and conductivity. Concentrations of ammonium and oxygen did not vary enough to explain the *amoA* gene and transcript dynamics. The observed herbivorous zooplankton may have caused a hidden flux of mineralized ammonium and a change in abundance of genes and transcripts. At the surface, AO might have been repressed during summer stratification due to nutrient limitation caused by active phytoplankton.

## 1. Introduction

Nitrogen cycling is one of the major biogeochemical processes on Earth. The discovery of novel nitrogen-converting pathways in the past decades [1] has shown the lack of knowledge we had and still have on global nitrogen cycling. Additionally, intensified use of fertilizers and nitrogenous precipitation derived from industry and traffic has led to large changes in the N-cycle in many ecosystems [2]. A major recent discovery in relation to the nitrification process was the role of Archaea in ammonia oxidation [3–5]. This notion has led to a great interest in the presence of ammonia-oxidizing archaea and bacteria in many ecosystems, often determined by the occurrence of archaeal and bacterial *amoA* genes (e.g., [6, 7]). In most analyses, the presence of archaeal* amoA* genes outnumbered those of bacteria by orders of magnitudes. What this means for the relative activities of both groups has only been investigated in a few environmental studies [8, 9].

The ecological importance of AOA and AOB has been determined in several studies; the relative abundance of AOA and AOB in soils is thought to be influenced mainly by pH [10, 11], temperature [12], and ammonium [13, 14], while in marine systems, next to ammonium [15], oxygen concentrations are expected to play a major role in the presence and abundance of AOA and AOB [16, 17]. However, studies comprising this type of analyses in relation to the occurrence of AOA and AOB in freshwater systems lag behind those related to terrestrial and marine studies.

The ecology of nitrifying bacteria in lakes is well described throughout the years (e.g., [18–21]), but the mutual presence of AOA and AOB was recorded only in some lakes and only at one time point. Lehours et al. [22, 23] found a different archaeal and bacterial community in oxic and permanent anoxic parts of monomictic Lake Pavin. In the sediment of the hypertrophic Lake Taihu, archaea dominated the prokaryotic community, likely due to the low oxygen conditions; no archaea could be detected in the water column [24–26]. In high-altitude Tibetan lakes, salinity influenced the abundance and community composition of AOA, which outnumbered AOB [27]. 

A first freshwater interannual analysis of Archaea showed the presence of a high diversity of thaumarchaeota (formerly thought to be part of the crenarchaeota phylum) in sulfurous karstic Lake Vilar, but only on the basis of the presence of the 16S rRNA gene [28]. These authors observed differences in richness distribution and seasonality, but no clear correlations were obtained when multivariate statistical analyses were carried out. No temporal comparison of both AOA and AOB in freshwater ecosystems has been made to date. 

Here we present a temporal and spatial study of the abundance of the *amoA* genes and the *amoA* gene transcripts as indicators of the presence and the status of activity, respectively, of AOB and AOA in the oligomictic Lake Lucerne. This lake, with high thaumarchaeota-specific crenarchaeol concentrations [29] and relatively high amounts of nitrogen [30], was expected to present a good site for studying the ecology of ammonia oxidizers (AO). The AOA and AOB have a similar temporal distribution pattern, though the AOA outnumber the AOB gene abundance at 42 m water depth and water just above the sediment. In the surface water the AO gene numbers were lower in the summer months, at which time the AOB outnumber the AOA, and a negative correlation of AOA with temperature and conductivity is found.

## 2. Materials and Methods

### 2.1. Location Description

Lake Lucerne is a perialpine lake located in Central Switzerland (47°N, 8°E; 434 m a.s.l) at the northern alpine front, with a catchment area of 2124 km^2^. It covers an area of 116 km^2^, contains seven basins, and is fed by four major alpine rivers (Reuss, Muota, Engelberger Aa, and Sarner Aa providing ~80% of the lakes total water supply (109 m^3^/s)) [31] with a 3.4-year residence time. As an oligomictic lake, a complete overturn occurs on average every six years. Sampling was done in the Kreuztrichter basin, one of the subbasins of Lake Lucerne, situated in the relatively open, western part of the lake.

### 2.2. Determination of Environmental Factors

Conductivity, temperature, oxygen, and pH were measured at the sampling location throughout the water column with a CTD scanner. 

The concentrations of ammonium, nitrate, and dissolved organic nitrogen (DON) were measured on a SEAL-QuAAtro autoanalyzer (Seal, Norderstedt, Germany). Detection limits were 0.16 *μ*mol for ammonium, 0.10 *μ*mol for nitrate, and 2 *μ*mol for DON. The concentration of dissolved organic carbon (DOC) was determined with a Formacs DOC analyzer (Skalar, Breda, The Netherlands) with a detection limit of 20 *μ*mol.

### 2.3. Sampling

Lake water was collected and filtered from the water surface (t = top, 0 m depth), the middle of the water column (m = middle, 42 m depth), and at the bottom, just above the sediment (b = bottom, varying from 72 m to 101 m depth due to slight location changes at different sampling times and the bathymetry at the sampling point in the Kreuztrichter basin) from January 2008 to April 2009. One sample was taken at each depth every month. Depending on the load of suspended particles, 1 to 3 liters of lake water were filtered. Samples for RNA analysis were frozen in a transportable liquid nitrogen freezer directly after filtration and stored at −80°C. 

### 2.4. Nucleic Acids Extractions

DNA was extracted as described previously [32]. In brief, cells were lysed by bead-beating followed by a phenol-cholorform-isoamyl alcohol extraction. The DNA was precipitated and dissolved in 100 mL of molecular biology grade water (Sigma-Aldrich, St. Louis, MO, USA). After extraction, the DNA was purified on a Wizard column (Promega, Madison, WI, USA) and the quantity of DNA was determined spectrophotometrically using a Nanodrop ND-1000 spectrophotometer (Nanodrop Technology, Wilmington, DE, USA).

RNA was extracted with an adjusted protocol of Culley et al. [33], in which one mL of Trizol was added to a tube containing half of a 47 mm 0.2 *μ*m pore-size membrane filter, over which a known amount of water was filtered (1.5 to 2 liters depending on the amount of suspended material) and followed by subsequent bead-beating and RNA isolation steps. RNA was purified from DNA using the Ambion Turbo DNA-free kit (Applied Biosystems, Austin, TX, USA) twice on each sample (as described by the manufacturer). DNA contamination was tested by performing PCR on the samples with primer sets F357 and R518 [34] for the 16S rRNA gene of bacteria.

The BioRad iScript kit with random hexamers (Bio-Rad Laboratories Inc., Hercules, CA, USA) was used to perform reverse transcriptase cDNA production.

### 2.5. Plankton Measurements

Abundances of planktonic organisms were determined by microscopy in a monthly monitor of a mixed sample of the upper 20 m of the Kreuztrichter basin and were kindly provided for this study by Dr. Hans-Rudolf Bürgi (Eawag). 

A principal component analysis on the presence of phyto- and zooplankton was made, in which the explanatory power of the abundance of these organisms on the AOA and AOB* amoA* gene abundances and diversities was established.

### 2.6. Clone Library Construction and Sequencing

Clone libraries of archaeal *amoA* genes were made of the water samples taken in December by the use of the pGEM-T Vector system (Promega, Madison, WI, USA). Hundred clones were processed and analyzed per water depth. Selected clones were sequenced with their amplification primers (Macrogen Inc., Republic of Korea) (Supplementary Table  2 of the Supplementary Material available online at http://dx.doi.org/10.1155/2013/289478).

### 2.7. Quantitative PCR of Archaeal and Bacterial *amoA* Genes

qPCR of archaeal and bacterial *amoA* genes was performed in a 20 *μ*L mixture of 10 *μ*L iQTM SYBR Green Supermix (Bio-Rad), 1 *μ*M of forward and reverse primers, and 0.2 mg mL-1 BSA. For archaeal standards, serial dilutions of the linearized soil fosmid clone 54d9 were used. For bacterial standards, a serial dilution of the linearized plasmid (pCR4-TOPO, Invitrogen) containing the *amoA* gene of *Nitrosomonas europaea* was used. For the archaeal *amoA* gene the forward primer 104(L)F (5′-GCAGGWGAYTACATYTTCTA-3′) was designed after the alignment of soil, marine, and freshwater clone sequences [14] and modified including and favoring clone sequences obtained from archaeal *amoA *genes found in Lake Lucerne sampled in December 2008 (Supplementary Table  2). Thus, the primer should be considered specific for *amoA* gene sequences dominating this lake. Amplifications were performed in Realplex (Mastercycler ep realplex, Eppendorf). Melting curve analyses were performed at the end of every qPCR run to confirm the amplification of the target products only, followed by standard agarose gel electrophoresis for affirmation. The following qPCR-program was used for both analyses—initial denaturation: 95°C for 15 minutes followed by 40 cycles of 95°C for 15 seconds, 55°C for 30 seconds, and 72°C for 40 seconds.

### 2.8. Statistical Analysis

Statistical analysis was performed using the Statistica 9 program (Statsoft Inc., Tulsa, OK, USA). The gene abundance was log-transformed to create normal distributions. A table of Spearman rank-order correlations of all variables was subsequently produced. A multiple-regression analysis and principal component analysis on the presence of phyto- and zooplankton and chemical compounds were made, in which the explanatory power of the concentrations of these compounds and organisms on the AOA and AOB* amoA* gene abundances and diversities was established.

## 3. Results and Discussion

### 3.1. Environmental Parameters

**Figure 1:**
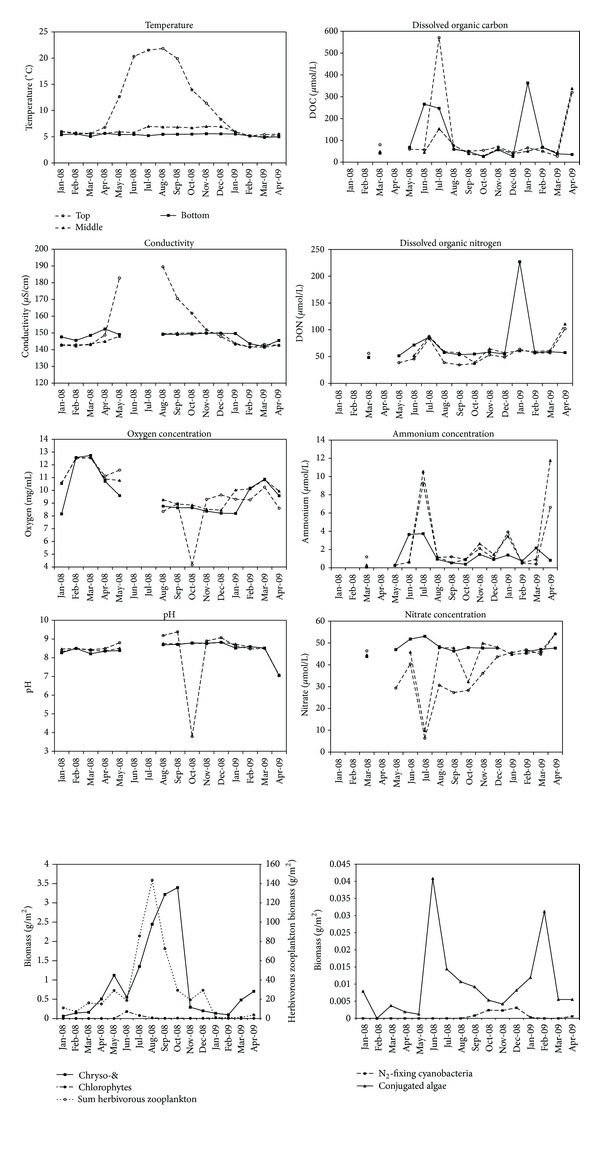
Temporal distribution of environmental factors at three water depths in Lake Lucerne. The single drop in pH and oxygen concentration in the surface water in October 2008 is expected to be caused by a failure of the equipment as such low pH values and oxygen concentrations are not observed in Lake Lucerne.

The oligotrophic nature of Lake Lucerne is reflected by an oxygenated water column with generally low nutrient levels, but with relatively high nitrogen concentrations in the form of nitrate (on average 63 *μ*mol/L) (Figure 1).

During our sixteen-month study, pH and oxygen did not vary at the different sampling depths of Lake Lucerne. More dynamic were the conductivity and temperature in the lake, especially in the surface water.

In July 2008 and April 2009, DOC and DON showed a peak at all depths, while in December 2008, DOC and DON peaked strongly in the water above the sediment, suggesting a more active decomposing microbial community at these times.

**Figure 2:**
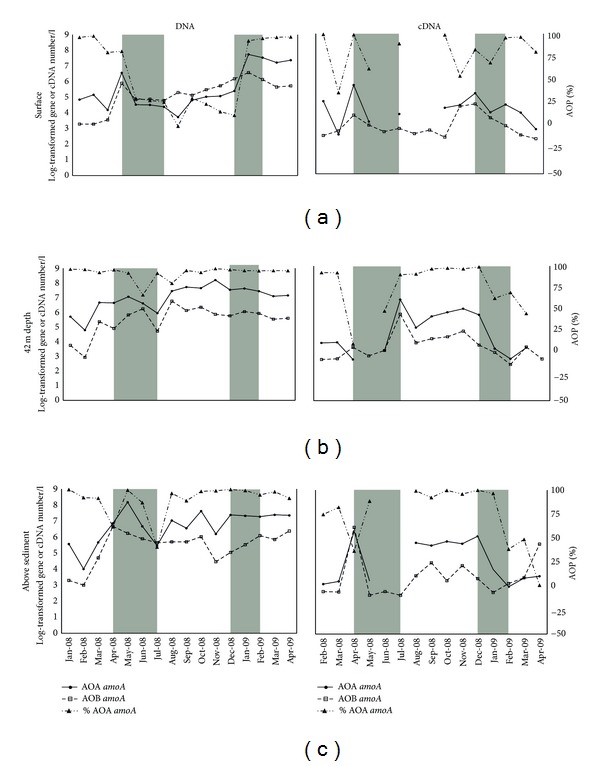
Temporal distribution of ammonia-oxidizing archaea (AOA) (solid lines, circles) and ammonia-oxidizing bacteria (AOB) (broken lines, squares), *amoA* gene abundances, and the archaeal percentage of the total *amoA* genes (broken line, triangles), all determined in three different layers in the water column of Lake Lucerne. In the left panels the DNA gene abundances are shown, on the right the cDNA abundances. Periods of mixing of the water layers are depicted by grey rectangles. Gene abundances were obtained by taking the average of three replicated qPCR analyses. Standard deviations of the replicates are indicated by error bars.

Ammonium concentrations were mostly around the detection level of 0.16 *μ*M but showed a peak in the surface water and at 42 m when nitrate showed a minimum. The opposing fluctuations of ammonium and nitrate concentrations may suggest that ammonia oxidation plays a role in Lake Lucerne, which is confirmed by low AOA and AOB abundances in the periods with high concentrations of ammonium and low concentrations of nitrate and vice versa (Figures 1 and 2).

### 3.2. AOA and AOB *amoA* Gene Numbers

The increase and decrease of AOA and AOB *amoA *gene abundances showed similar patterns among the sixteen monthly collected samples at all three depths, indicating that AOA and AOB are generally displaying similar population dynamics (Figure 2). This observation is supported by significant (*P* < 0.05) and positive Spearman rank-order correlations between the gene copy numbers (Supplementary Table  1).

An increase in abundance of both AOA and AOB was observed in March (surface) and April (deeper waters) 2008, with the onset of summer stratification in the water column of Lake Lucerne, and an increase in AO was again observed in December 2008 when the water layers mixed again. During the period of water stratification, the numbers of AOA at the surface declined more than those of AOB leading to a lower percentage of the total AO of the first one. This period of lower AO numbers and AOB dominance at the surface of the lake coincided with relatively warm water and a higher conductivity (Figure 1). When comparing the gene copy numbers obtained in the summer stratification period, that is, from June till September, for which ANOVA pointed to a different temperature compared with the rest of the sampling period, it appears that the means of the archaeal gene copy numbers obtained in these two periods were only significantly different in the surface water (Table 1). With bacterial gene copy numbers, no significant differences between the means were observed throughout the water column. Water depth did also not significantly affect the AOB *amoA *gene abundance in the water column of the lake. In contrast, the AOA *amoA* gene abundance increased from the surface to the deeper water layers, giving rise to an increasing AOA/AOB ratio with depth, which is also observed in other aquatic systems [35–37].

We observed (Figure 2) and confirmed by one-way ANOVA that AOA in the surface water behaved differently from the AOA in the deeper waters (*P* < 0.005), which was not observed for AOB (*P* < 0.6). This all suggests that the low AOA/AOB ratio at the surface water is caused by an environment in which different AOA dynamics or even communities occur compared to waters at greater depth.

The most striking result of our temporal study was the generally similar behavior of the archaeal and bacterial ammonia-oxidizing communities through time, suggesting a situation in which AOA and AOB cooccur rather than compete for nutrients. 

### 3.3. AOA and AOB *amoA* Gene Transcript Numbers

On the cDNA level, the differences between the two domains were even less pronounced (Figure 2, right panels). The transcripts of the *amoA *genes also showed mutual temporal dynamics and higher abundances in the water column at the onset and end of summer stratification, except in the middle of the water column, where the transcripts were most abundant during summer stratification. Higher gene transcript numbers at moments before and after stratification are likely due to mixing of the water column and subsequent increased nutrient availability leading to higher metabolic activities [38–40].

Generally, an increased *amoA *cDNA level was observed a month before or at the same time of a rise in *amoA *genes, suggesting a higher ammonia-oxidizing activity when cells started to multiply (Figure 2). This was, however, less clear for the surface layer of the water column, where cDNA was even below the detection limit in the months in which the numbers of the *amoA *gene of AOB exceeded those of the AOA. Hence, not only cell numbers of AOA were lower then, but also the transcription activity was undetectable for AOA. In the surface water in December 2008, however, when the AOA outnumbered the AOB once more, the amount of archaeal *amoA*-related cDNA had the highest increase rate, as one would expect at moments of population growth. 

### 3.4. AOA and AOB *amoA* Genes and Transcripts in relation to Environmental Factors

**Figure 3:**
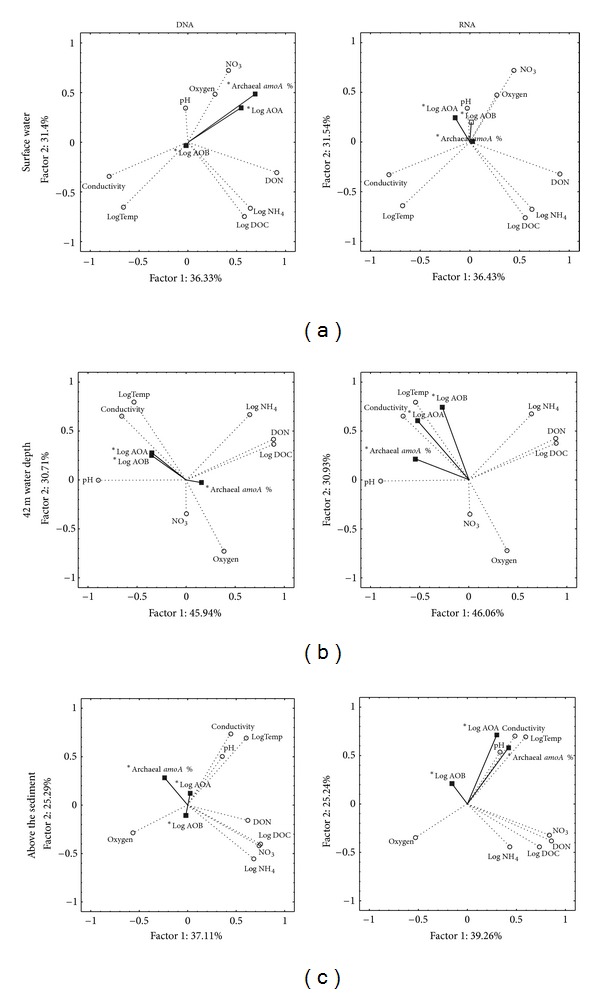
Principal component analysis of ammonia oxidizers DNA (left) and RNA (right) and environmental factors and nutrients in the surface water (above), 42 m water depth (middle), and water just above the sediment (below). A principal component analysis on chemical compounds was made, in which the explanatory power of the concentrations of these compounds on the AOA and AOB* amoA* gene abundances and diversities was established. Statistical analysis was performed using the Statistica 9 program (Statsoft Inc., Tulsa, OK, USA).

Different environmental factors correlated to AOA and AOB *amoA* genes and transcripts throughout seasons and depths, as is shown by Spearman rank-order correlation analysis (Supplementary Table  1) and supported and visualized by PCA analysis (Figure 3). The main environmental factors influencing the AOA populations in previous studies, that is, pH, ammonium concentration, and oxygen availability, showed little dynamics in our study site; hence little influence on the AO gene and transcript abundances could be assigned to these factors. Additionally, the factors that showed the strongest explanatory power in our study, that is, temperature and conductivity, were constant throughout the season in the deeper water layers, opposite to the changes observed for the surface water. When considering all water depths of Lake Lucerne, conductivity explained 53% of the variance in the distribution of AOA. Conductivity was also of great influence on AO dispersal in Tibetan lakes [27], where lake biochemistry seemed to shape the archaeal community rather than historic events.

Conductivity in the Kreuztrichter basin was described to be affected by processes that are connected to phytoplankton dynamics, such as carbon assimilation, calcite precipitation, sedimentation, and decomposition in the hypolimnion [41]. A change in conductivity therefore may reflect a change in local nutrient availability due to phytoplankton activity, which probably affects the dynamics of AOA and AOB, though each in a specific manner as revealed by ANOVA (Table 1).

The concentration of ammonium, the expected substrate, was mostly around the detection limit and no relation with the transcript abundance of the functional gene for ammonia-oxidation could be found. The nitrate concentration in Lake Lucerne is expected to change by biochemical cycling only, as the inflow of fresh water is limited and originates from other basins of the Lake, rather than from the surrounding catchment. However, nitrate, the endproduct of nitrification, did not correlate with bacterial *amoA* genes or gene transcript abundances neither with archaeal *amoA* transcripts. Nitrate did however correlate with archaeal *amoA* gene abundance, but only in the surface water. To date the comparisons of AOA and AOB ammonium uptake kinetics are based on a limited number of pure culture experiments, and so far it is unknown if AOA and AOB in natural environments behave similarly. AOA were found to thrive at low nutrient concentrations [42] and showed growth until ammonium concentrations fell below the detection level (i.e., 10 nM), which is a 100-fold lower than the threshold concentration for AOB (1 *μ*M at near neutral pH) [15]. In accordance with these findings, ammonium was generally around the detection limit in the waters of our study site, where AOB only reached low cell numbers (Figures 1 and 2) and were outnumbered by AOA by 1 or 2 orders of magnitude difference in gene abundance in the deeper waters. Also in the North Sea, a similar temporal dynamic of AOA and AOB was observed with AOA outnumbering AOB by 1 or 2 orders of magnitude [3], suggesting this might be more common in aquatic environments.

In the surface water the abundance of AOB was higher than that of AOA during summer stratification when temperature and conductivity increased (Figures 1 and 2); this is due to a negative correlation of AOA with conductivity and temperature, rather than a positive correlation of AOB with these factors. However, temperature and conductivity correlated positively with cDNA derived from archaeal and bacterial *amoA* in the deeper layers, although for the bacterial cDNA only at 42 m depth. Apparently, temperature and conductivity stimulated the transcription activity of the ammonia oxidizers in the deeper layers, but not in the surface water. Hence, some other factor must have been responsible for the relative increase of AOB in relation to AOA in the surface layer during summer stratification.

It has been suggested that oxygen influences the composition of AOB communities [43] and low oxygen levels may offer a niche for AOA [16, 17, 44–46]. However, since the concentration of oxygen varied only little at the different water depths of the well-oxygenated water column of Lake Lucerne, oxygen is not likely to be a selective environmental factor with respect to the presence of AOA and AOB in lake Lucerne. 

### 3.5. Correlation of AO Genes and Gene Transcript Numbers to the Presence of Other Plankton

**Figure 4:**
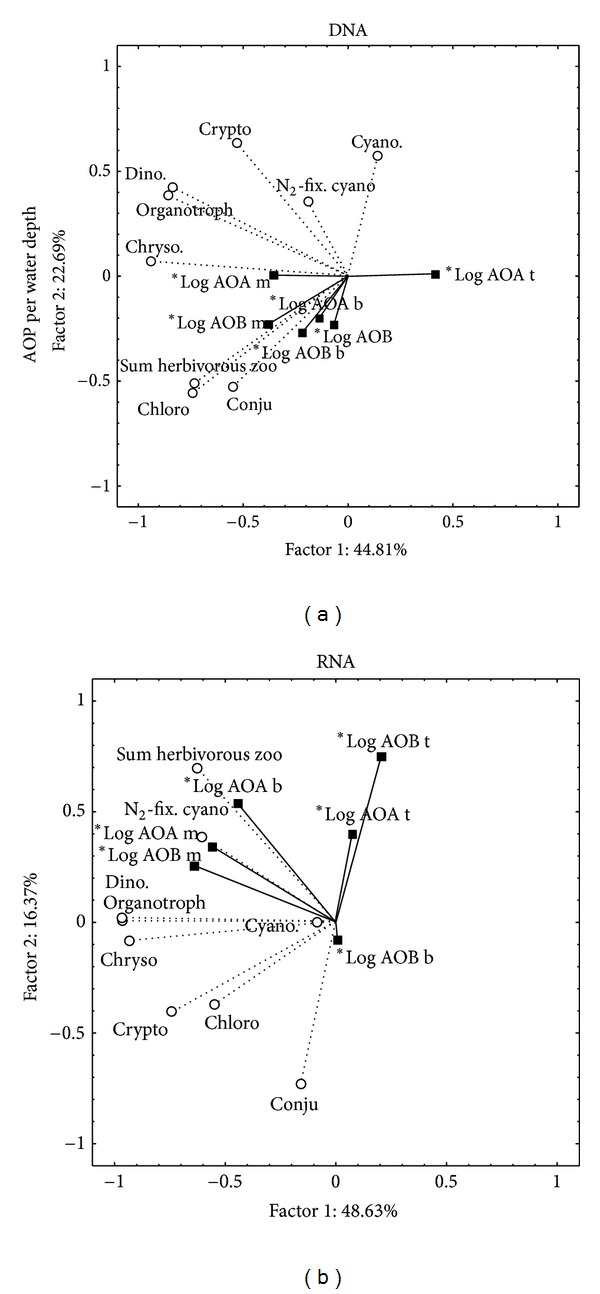
Principal component analysis of ammonia oxidizers DNA (a) and RNA (b) and other planktonic microorganisms abundance in the Kreuztrichter basin, Lake Lucerne. Ammonia oxidizers' abundances are printed per water depth on the representation of the abundance of the other planktonic organisms observed throughout the upper 20 m of the water column.

AOA *amoA* genes and gene transcripts in deeper waters, as well as AOB *amoA *transcripts throughout the water column, correlated to numbers of herbivorous zooplankton and N_2_-fixing cyanobacteria (Figure 4). These plankton groups may supply AOA and AOB directly or indirectly with extra ammonium from mineralization of organic nitrogen compounds. Correlations with herbivorous and mixotrophic zooplankton were found in all water depths. A possible explanation for increasing amounts of *amoA* transcripts might be the increase of activity during grazing. It has been shown in ammonia-limited chemostats containing pure cultures of AOB and heterotrophic bacteria that grazing by a flagellate lowered the number of ammonia-oxidizing cells present in the culture, but increased at the same time the oxidation rate per cell [47]. AOB cells have a higher amount of mRNA ready for ammonia oxidation at moments before growth is observed, which possibly causes the AOB population to recover faster after predation, while the AOA population needs more time to recover from phagotrophy.

In the surface water, a negative correlation was observed between AOA gene and gene transcript numbers on one side, and the numbers of conjugate algae and chrysophytes on the other side. Chrysophytes are described to be mixotrophs as they obtain energy either from light or by feeding on decaying or living cells [48]. This predation could cause the decline of the archaeal and bacterial cell numbers in the surface water during summer stratification, when the chrysophyte bloom was observed. 

An explanation for the lower numbers of AO in the surface water may be found in surface-related factors such as a competition with phototrophic microorganisms for nutrients and CO_2_ or an inhibition by light. The community of AOB in the surface water is apparently less affected than the AOA by these factors from May till December 2008. Outside this period of summer stratification the negative factors for the AOA in the surface layer seem to be less severe, which might lead again to their dominance. More research is required to elucidate this differential effect of surface water factors on AOA and AOB. 

## 4. Conclusions

The low availability of ammonium in the lake throughout the year may favor AOA over AOB leading to larger population sizes of the first group [49]. Although with different amplitudes, AOA and AOB followed more or less the same temporal changes throughout the water column. Assuming that they have to compete for the same resource, a similarity in community dynamics between archaeal and bacterial ammonia-oxidizing microorganisms is not expected. Even chaotic behavior of pelagic populations makes such a similarity in temporal dynamics not likely [50]. This either means that the amount of ammonium was not limiting or that AOA can utilize other resources next to ammonium, as has been suggested by [51]. Increase in gene and gene transcript abundance cooccurred with the mixing of the water column before and after summer stratification in the lake, which may indicate a rapid response to changing conditions such as ammonium availability. In Lake Lucerne, ammonium levels were mostly very low. However, ammonium could be available as a nutrient for AOA and AOB by direct local production, which was supported by the observation that AOB and AOA in the deeper waters correlated to herbivorous zooplankton, which make ammonium available by their grazing activity. In the surface water, UV inhibition as well as predation and competition for nutrients and CO_2_ by zooplankton may have influenced the population size of the AOP negatively. In addition, not only the size of the AOA community based on both the abundance of the *amoA* gene and that of the 16S rRNA gene was significantly affected in the surface layer by factors prevailing during the period of summer stratification, but also the diversity of the dominant strains as appearing from DGGE profiling of the *amoA* gene [52] was significantly affected in this period (Table 1).

## Supplementary Material

Supplementary Table 1: Spearman rank order correlation coefficients of biotic and abiotic factors determined in the water column of Lake Lucerne at three different depths. Shown are the values that are statistically significant (*P* <0.05; DNA *n* = 16, RNA *n* =15). Gene abundances were obtained by qPCR and were log-transformed to create normal distributions.
Supplementary Table 2: List of primers used in this study.

## Figures and Tables

**Table 1:** One-way ANOVA on the differences between the means of community characteristics of ammonia-oxidizing archaea and bacteria determined for summer and winter months, respectively. The difference is significant when *F *_measured_ < *F *_critical_ and *F *_critical_ = 4.8443357. Significant differences are shown in bold. Individual data have been presented by Vissers et al. (in press).

Parameter	Water depth	*F*_ measured_	*P*
	Surface	**5.048039**	**0.04615**
Log archaeal 16S	−42 m	0.118078	0.73761
	Above sediment	0.708741	0.41779

	Surface	**7.093356**	**0.02205**
Log archaeal *amoA *	−42 m	0.061664	0.82351
	Above sediment	0.052174	0.41779

	Surface	0.020078	0.88988
Log bacterial *amoA *	−42 m	1.228674	0.29131
	Above sediment	0.622908	0.44665

	Surface	2.678394	0.12998
Number of archaeal 16S rRNA DGGE bands	−42 m	0.206905	0.65805
	Above sediment	0.151504	0.70453

	Surface	**29.27228**	**0.00021**
Number of archaeal *amoA* DGGE bands	−42 m	2.873572	0.11813
	Above sediment	2.925275	0.11522

## References

[1] Jetten MSM (2008). The microbial nitrogen cycle. *Environmental Microbiology*.

[2] Gruber N, Galloway JN (2008). An Earth-system perspective of the global nitrogen cycle. *Nature*.

[3] Wuchter C, Abbas B, Coolen MJL (2006). Archaeal nitrification in the ocean. *Proceedings of the National Academy of Sciences of the United States of America*.

[4] Könneke M, Bernhard AE, De La Torre JR, Walker CB, Waterbury JB, Stahl DA (2005). Isolation of an autotrophic ammonia-oxidizing marine archaeon. *Nature*.

[5] Treusch AH, Leininger S, Kietzin A, Schuster SC, Klenk HP, Schleper C (2005). Novel genes for nitrite reductase and Amo-related proteins indicate a role of uncultivated mesophilic crenarchaeota in nitrogen cycling. *Environmental Microbiology*.

[6] Francis CA, Roberts KJ, Beman JM, Santoro AE, Oakley BB (2005). Ubiquity and diversity of ammonia-oxidizing archaea in water columns and sediments of the ocean. *Proceedings of the National Academy of Sciences of the United States of America*.

[7] Rotthauwe JH, Witzel KP, Liesack W (1997). The ammonia monooxygenase structural gene *amoA* as a functional marker: molecular fine-scale analysis of natural ammonia-oxidizing populations. *Applied and Environmental Microbiology*.

[8] Nicol GW, Leininger S, Schleper C, Prosser JI (2008). The influence of soil pH on the diversity, abundance and transcriptional activity of ammonia oxidizing archaea and bacteria. *Environmental Microbiology*.

[9] Di HJ, Cameron KC, Shen JP (2009). Nitrification driven by bacteria and not archaea in nitrogen-rich grassland soils. *Nature Geoscience*.

[10] Nicol GW, Webster G, Glover LA, Prosser JI (2004). Differential response of archaeal and bacterial communities to nitrogen inputs and pH changes in upland pasture rhizosphere soil. *Environmental Microbiology*.

[11] He J-Z, Shen J-P, Zhang L-M (2007). Quantitative analyses of the abundance and composition of ammonia-oxidizing bacteria and ammonia-oxidizing archaea of a Chinese upland red soil under long-term fertilization practices. *Environmental Microbiology*.

[12] Tourna M, Freitag TE, Nicol GW, Prosser JI (2008). Growth, activity and temperature responses of ammonia-oxidizing archaea and bacteria in soil microcosms. *Environmental Microbiology*.

[13] Lehtovirta-Morley LE, Stoecker K, Vilcinskas A, Prosser JI, Nicol GW (2011). Cultivation of an obligate acidophilic ammonia oxidizer from a nitrifying acid soil. *Proceedings of the National Academy of Sciences of the United States of America*.

[14] Tourna M, Stieglmeier M, Spang A (2011). Nitrososphaera viennensis, an ammonia oxidizing archaeon from soil. *Proceedings of the National Academy of Sciences of the United States of America*.

[15] Martens-Habbena W, Berube PM, Urakawa H, De La Torre JR, Stahl DA (2009). Ammonia oxidation kinetics determine niche separation of nitrifying Archaea and Bacteria. *Nature*.

[16] Lam P, Lavik G, Jensen MM (2009). Revising the nitrogen cycle in the Peruvian oxygen minimum zone. *Proceedings of the National Academy of Sciences of the United States of America*.

[17] Coolen MJL, Abbas B, Van Bleijswijk J (2007). Putative ammonia-oxidizing Crenarchaeota in suboxic waters of the Black Sea: a basin-wide ecological study using 16S ribosomal and functional genes and membrane lipids. *Environmental Microbiology*.

[18] Laanbroek HJ, Bollmann A, Ward BB, Klotz MG, Arp DJ (2011). Nitrification in inland water. *Nitrification*.

[19] Coci M, Bodelier PLE, Laanbroek HJ (2008). Epiphyton as a niche for ammonia-oxidizing bacteria: detailed comparison with benthic and pelagic compartments in shallow freshwater lakes. *Applied and Environmental Microbiology*.

[20] Whitby CB, Saunders JR, Pickup RW, McCarthy AJ (2001). A comparison of ammonia-oxidiser populations in eutrophic and oligotrophic basins of a large freshwater lake. *Antonie van Leeuwenhoek, International Journal of General and Molecular Microbiology*.

[21] Vincent WF, Downes MT (1981). Nitrate accumulation in aerobic hypolimnia—relative importance of benthic and planktonic nitrifiers in an oligotrophic lake. *Applied and Environmental Microbiology*.

[22] Lehours AC, Bardot C, Thenot A, Debroas D, Fonty G (2005). Anaerobic microbial communities in Lake Pavin, a unique meromictic lake in France. *Applied and Environmental Microbiology*.

[23] Lehours AC, Evans P, Bardot C, Joblin K, Gérard F (2007). Phylogenetic diversity of archaea and bacteria in the anoxic zone of a meromictic lake (Lake Pavin, France). *Applied and Environmental Microbiology*.

[24] Liu L, Peng Y, Zheng X, Xiao L, Yang L (2010). Vertical structure of bacterial and archaeal communities within the sediment of a eutrophic lake as revealed by culture-independent methods. *Journal of Freshwater Ecology*.

[25] Wu Y, Xiang Y, Wang J, Zhong J, He J, Wu QL (2010). Heterogeneity of archaeal and bacterial ammonia-oxidizing communities in Lake Taihu, China. *Environmental Microbiology Reports*.

[26] Ye W, Liu X, Lin S (2009). The vertical distribution of bacterial and archaeal communities in the water and sediment of Lake Taihu. *FEMS Microbiology Ecology*.

[27] Hu A, Yao T, Jiao N, Liu Y, Yang Z, Liu X (2010). Community structures of ammonia-oxidising archaea and bacteria in high-altitude lakes on the Tibetan Plateau. *Freshwater Biology*.

[28] Llirós M, Casamayor EO, Borrego C (2008). High archaeal richness in the water column of a freshwater sulfurous karstic lake along an interannual study. *FEMS Microbiology Ecology*.

[29] Blaga CI, Reichart GJ, Heiri O, Sinninghe Damsté JS (2009). Tetraether membrane lipid distributions in water-column particulate matter and sediments: a study of 47 European lakes along a north-south transect. *Journal of Paleolimnology*.

[30] Bürgi HR, Stadelmann P (2002). Alteration of phytoplankton structure in Lake Lucerne due to trophic conditions. *Aquatic Ecosystem Health*.

[31] Schnellmann M, Anselmetti FS, Giardini D, McKenzie JA, Ward SN (2002). Prehistoric earthquake history revealed by lacustrine slump deposits. *Geology*.

[32] Vissers EW, Bodelier PLE, Muyzer G, Laanbroek HJ (2009). A nested PCR approach for improved recovery of archaeal 16S rRNA gene fragments from freshwater samples. *FEMS Microbiology Letters*.

[33] Culley DE, Kovacik WP, Brockman FJ, Zhang W (2006). Optimization of RNA isolation from the archaebacterium Methanosarcina barkeri and validation for oligonucleotide microarray analysis. *Journal of Microbiological Methods*.

[34] Muyzer G, De Waal EC, Uitterlinden AG (1993). Profiling of complex microbial populations by denaturing gradient gel electrophoresis analysis of polymerase chain reaction-amplified genes coding for 16S rRNA. *Applied and Environmental Microbiology*.

[35] Kirchman DL, Elifantz H, Dittel AI, Malmstrom RR, Cottrell MT (2007). Standing stocks and activity of Archaea and Bacteria in the western Arctic Ocean. *Limnology and Oceanography*.

[36] Callieri C, Corno G, Caravati E, Rasconi S, Contesini M, Bertoni R (2009). Bacteria, Archaea, and Crenarchaeota in the epilimnion and hypolimnion of a deep holo-oligomictic lake. *Applied and Environmental Microbiology*.

[37] Tamburini C, Garel M, Al Ali B (2009). Distribution and activity of Bacteria and Archaea in the different water masses of the Tyrrhenian Sea. *Deep-Sea Research Part II*.

[38] Winder M (2009). Photosynthetic picoplankton dynamics in Lake Tahoe: temporal and spatial niche partitioning among prokaryotic and eukaryotic cells. *Journal of Plankton Research*.

[39] Naiman R, Magnuson JJ, McKnight DM, Stanford JA (1995). *The Freshwater Imperative: A Research Agenda*.

[40] Fietz S, Kobanova G, Izmesteva L, Nicklisch A (2005). Regional, vertical and seasonal distribution of phytoplankton and photosynthetic pigments in Lake Baikal. *Journal of Plankton Research*.

[41] Bührer H, Ambühl H (2001). Lake Lucerne, Switzerland, a long term study of 1961–1992. *Aquatic Sciences*.

[42] Erguder TH, Boon N, Wittebolle L, Marzorati M, Verstraete W (2009). Environmental factors shaping the ecological niches of ammonia-oxidizing archaea. *FEMS Microbiology Reviews*.

[43] Bollmann A, Laanbroek HJ (2002). Influence of oxygen partial pressure and salinity on the community composition of ammonia-oxidizing bacteria in the Schelde estuary. *Aquatic Microbial Ecology*.

[44] Beman JM, Popp BN, Francis CA (2008). Molecular and biogeochemical evidence for ammonia oxidation by marine Crenarchaeota in the Gulf of California. *ISME Journal*.

[45] Lam P, Jensen MM, Lavik G (2007). Linking crenarchaeal and bacterial nitrification to anammox in the Black Sea. *Proceedings of the National Academy of Sciences of the United States of America*.

[46] Yan J, Haaijer SCM, Op den Camp HJM (2012). Mimicking the oxygen minimum zones: stimulating interaction of aerobic archaeal and anaerobic bacterial ammonia oxidizers in a laboratory-scale model system. *Environmental Microbiology*.

[47] Verhagen FJM, Laanbroek HJ (1991). Competition for ammonium between nitrifying and heterotrophic bacteria in dual energy-limited chemostats. *Applied and Environmental Microbiology*.

[48] Holen DA, Boraas ME (1995). Mixotrophy in chrysophytes. *Chrysophyte Algae Ecology, Phylogeny and Development*.

[49] Schleper C, Nicol GW (2010). Ammonia-oxidising archaea—physiology, ecology and evolution. *Advances in Microbial Physiology*.

[50] Huisman J, Weissing FJ (1999). Biodiversity of plankton by species oscillations and chaos. *Nature*.

[51] Blainey PC, Mosier AC, Potanina A, Francis CA, Quake SR (2011). Genome of a low-salinity ammonia-oxidizing archaeon determined by single-cell and metagenomic analysis. *PLoS ONE*.

[52] Vissers EW, Blaga CI, Bodelier PLE (2013). Seasonal and vertical distribution of putative ammonia-oxidizing thaumarchaeotal communities in an oligotrophic lake. *FEMS Microbiology Ecology*.

