# Supplementary material for: Temporal and Spatial Coexistence of Archaeal and Bacterial amoA Genes and Gene Transcripts in Lake Lucerne
Source: Archaea. 2013 Mar 5;2013:289478. doi: 10.1155/2013/289478 (PMC3603158; doi:10.1155/2013/289478)
Supplement: Supplementary file 2 [file 289478.f2.docx]

|  | **target** | **primer name** | **sequence** | **reference** |
| --- | --- | --- | --- | --- |
| **qPCR** | Archaeal *amoA* | 104(L)F | 5´-GCAGGWGAYTACATYTTCTA-3´ | (Vissers et al, submitted for publication) |
| (1µM primer used) |  | 616R | 5'- GCCATCCATCTGTATGTCCA - 3' | ([Tourna*, et al.*, 2008](#_ENREF_4)) |
|  | Bacterial *amoA* | 1F(J) | 5'- GGGGHTTYTACTGGTGGT - 3' | ([Stephen*, et al.*, 1999](#_ENREF_3)) |
|  |  | 2R | 5'- CCCCTCKGSAAAGCCTTCTTC - 3' | ([Rotthauwe*, et al.*, 1997](#_ENREF_2)) |
|  |  |  |  |  |
| **Clone library** | Archaeal *amoA* | AOA-*amo*A-F | 5′-STAATGGTCTGGCTTAGACG-3′ | ([Francis*, et al.*, 2005](#_ENREF_1)) |
| (0.5 µM primer used) |  | AOA-amoA-R | 5′-GCGGCCATCCATCTGTATGT-3′ | ([Francis*, et al.*, 2005](#_ENREF_1)) |
